# Lactic acid fermentation of guava juice: Evaluation of nutritional and bioactive properties, enzyme (α‐amylase, α‐glucosidase, and angiotensin‐converting enzyme) inhibition abilities, and anti‐inflammatory activities

**DOI:** 10.1002/fsn3.3683

**Published:** 2023-09-14

**Authors:** Seyed Mohammad Bagher Hashemi, Dornoush Jafarpour

**Affiliations:** ^1^ Department of Food Science and Technology, Faculty of Agriculture Fasa University Fasa Iran; ^2^ Department of Food Science and Technology, Faculty of Agriculture, Fasa Branch Islamic Azad University Fasa Iran

**Keywords:** bioactive compounds, fermentation, guava juice, health properties

## Abstract

In the present research, the impact of fermentation with two strains of *Lactiplantibacillus plantarum* subsp. *plantarum* (PTCC 1896 and PTCC 1745) on physicochemical properties, antioxidant bioactive compounds, and some health‐promoting features of guava juice was investigated. Results showed a significant (*p <* .05) decrease in pH, total soluble solids, glucose and fructose residues, vitamin C, and total carotenoids after 32 h of fermentation. Total phenolic content, free radical scavenging abilities, and ferrous reducing power were markedly enhanced during the fermentation process. Moreover, fermented juice represented good enzyme inhibition abilities (α‐amylase and α‐glucosidase) and anti‐inflammatory activities. The initial amount of angiotensin‐converting enzyme inhibitory activity (26.5%) increased to 72.1% and 66.4% in *L. plantarum* subsp. *plantarum* 1896 and *L. plantarum* subsp. *plantarum* 1745 treatments, respectively. These findings reveal that guava juice fermentation with the studied *Lactobacillus* strains can be a promising strategy to augment the functional properties of the fruit‐based beverage.

## Graphical abstract

In the present study, the functional properties of guava juice fermented by Lactiplantibacillus plantarum were investigated. In this regard, some features of the fermented beverage including antioxidant activity, α‐amylase and α‐glucosidase inhibition abilities, angiotensin‐converting enzyme (ACE) inhibition, and anti‐inflammatory activities were determined.

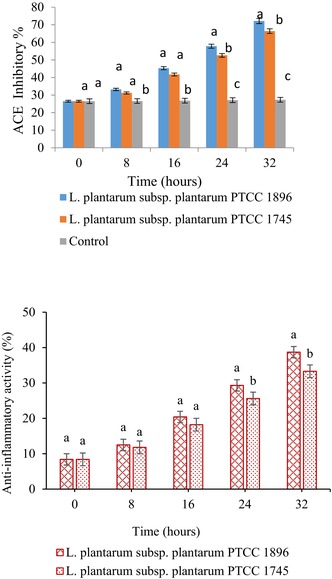

## 1 INTRODUCTION

Guava is one of the most important commercial fruits in subtropical and tropical regions, which belongs to the Myrtaceae family and *Psidium* genus called *Psidium guajava L*. (Gull et al., 2012). This plant is native to America, and today its cultivation has spread to other parts of the world, including southwestern Europe, India, and Australia (Upadhyay et al., 2019). This fruit has an interesting nutritional profile characterized by a low content of proteins, fats, and carbohydrates and high content in minerals (e.g., potassium, phosphorus, and calcium), vitamins (e.g., A, niacin, and C), as well as dietary fibers. For instance, it is reported that a guava has 4–5 times more vitamin C than a single orange (Lamo et al., 2019), being considered one of the richest sources of antioxidants among tropical fruits. Moreover, it contains numerous biologically active compounds such as triterpenoids, phenolic acids, and flavonoids, having anti‐inflammatory, antiplasmodial, anticancer, and hepato‐protective effects (Flores et al., 2015).

Fermentation is a set of biological activities by microorganisms or their enzymes in which carbohydrates and other related compounds are converted into organic compounds. Generally, fermentation improves nutritional quality, increases bioactive compounds, enhances antioxidant activity, and changes chemical compounds (Hashemi et al., 2021). Lactic acid bacteria (LAB) are among the microorganisms that play an essential role in the food industry due to their fermentation capability. Fermentation with LAB not only affects the organoleptic properties of the final product but also positively improves the bioactive compounds and antioxidant properties of the fermentation substrate (Zhang et al., 2021). In addition, fermentation with LAB has been shown to have health‐promoting effects. In this line, the antioxidant and anti‐inflammatory activities of different beverages (i.e., peach juice, orange juice‐milk. etc.) have been increased during the fermentation process by *Lactobacillus* strains (de la Fuente et al., 2021; Hashemi et al., 2021).

Recently, the demand for nondairy products has increased, with the increase in the number of vegetarians and people with lactose intolerance. One of the recent activities in this field is producing nondairy products fermented by LAB based on fruit and vegetable juices (Hashemi & Jafarpour, 2020). Besides being a rich source of nutrients, fruit juices are a suitable substrate for fermentation. The carbon sources in fruit juices can promote the growth of cultures and facilitate the production of new flavor profiles (Ayed et al., 2020). Several studies have demonstrated the application of *Lactobacillus* strains in the fermentation of fruit juices and the production of nondairy fermented beverages (Kwaw et al., 2018; Liao et al., 2016; Mousavi et al., 2013). Considering the beneficial impacts of fermented products and few studies on guava juice fermentation, it was decided to use two *Lactobacillus* strains to produce a nondairy fermented beverage. Hence, the aim of this study was to evaluate the functional properties of guava juice fermented by *L. plantarum* subsp. *plantarum* PTCC 1896 and *L. plantarum* subsp. *plantarum* PTCC 1745. In this regard, some features of the fermented beverage, including antioxidant activity, α‐amylase, and α‐glucosidase inhibition abilities, angiotensin‐converting enzyme (ACE) inhibition, and anti‐inflammatory activities, were investigated.

## 2 MATERIALS AND METHODS

### 2.1 Juice preparation

Guava fruits at the ripening stage were purchased from a local shop in Bandar Abbas. The fruits were selected for homogeny in size, maturity, and freedom from defects. The fruits were cleaned and sanitized in sodium hypochlorite solution at 120 ppm for 15 min and wiped dry at ambient temperature. The fruits were cut into pieces and squeezed by a juicer to prepare guava juice (Pars Khazar, JBG‐610P). The juice was then filtered through a cotton cloth to obtain clear juice. The clear juice was pasteurized by a water bath at 90°C for 5 min.

### 2.2 Microorganisms, inoculum preparation, and fermentation

*L. plantarum* subsp. *plantarum* PTCC 1896 and *L. plantarum* subsp. *plantarum* PTCC 1745 were purchased from Iranian Research Organization for Science and Technology. The strains were reactivated in the MRS broth (Oxoid) at 37°C for 24 h. Cell pellets were collected by centrifugation (Hettich; 3500 *g*, 15 min, 4°C), washed thrice, and collected again by centrifugation. The strains were inoculated separately to the juice samples (~5.3 log CFU/mL; 200 mL) in sterile Erlenmeyer flasks and subjected to fermentation at 37°C for 32 h. The control samples were the samples without LAB.

### 2.3 Enumeration of *Lactobacillus* strains during fermentation

A 1‐mL aliquot of each fermentation sample was diluted with peptone water and 0.1 mL of aliquots of these dilutions were plated on MRS agar (Oxoid) (Hashemi & Jafarpour, 2020). After an incubation time at 37°C for 72 h, the observable colonies were counted, and results were reported as log CFU/mL.

### 2.4 Physicochemical properties

The pH values were determined using a digital pH meter (Metrohm 744, Swiss). The acid content was measured by titrating samples with 0.1 N NaOH, and total soluble solids (TSS, °Brix) were determined using a digital refractometer (Atago Rx‐5000, Japan) (Agza et al., 2018).

### 2.5 HPLC measurement of glucose and fructose

Glucose and fructose were determined using HPLC (Knauer) with a K‐2310 refractive index (RI) detector. Eurokat H (250 × 30 mm) was utilized as a separation column. Sulfuric acid (2.25 mM) was employed as a mobile phase with 0.4 mL/min flow rate at 45°C. The injection volume was 20 μL (Mousavi et al., 2013).

### 2.6 HPLC determination of vitamin C

Vitamin C of guava juice samples was determined by an HPLC (Knauer, Azura) with a C18 column (15 cm × 4.6 cm, pore size 5 μm). For injection, 25 mL of juice sample was supplemented with 5 mL of metaphosphoric acid (2.5%), and afterward, 10 μL of the obtained solution was used. The mobile phase was KH_2_PO_4_ (0.025 M) with 1 mL/min flow rate. An ultraviolet–visible detector (UVD2.1 L) was employed to monitor the eluate at 245 nm (Hashemi et al., 2019).

### 2.7 Antioxidant and bioactive assays

For total carotenoids measurement, a 25‐mL aliquot of guava juice was homogenized with 50 mL of extracting solvent (hexane/acetone/ethanol, 50:25:25, v/v/v), and centrifugation was done for 10 min at 3000 *g*. The top layer of hexane was recovered, and absorbance at 450 nm using a spectrophotometer (Shimadzu UV‐1601PC) was determined (Lee & Castle, 2001).

To determine total phenolic compounds (TPC), briefly, 0.1 mL of Folin Ciocalteu's phenol reagent (0.2 N) was mixed with 1‐mL guava juice sample and placed at ambient temperature for 5 min. Subsequently, it was supplemented with 0.2 mL Na_2_CO_3_ solution (7.5%) and vortexed for 2 min. Then, the solution was kept at 25°C for 1 h. The distilled water was used as a blank, and the absorbance was read at 765 nm by a spectrophotometer (Kwaw et al., 2018).

For radical scavenging activity, 1‐diphenyl‐2‐picrylhydrazyl (DPPH) solution (0.1 mmol/L) was prepared with methanol, and 100‐μL juice sample was supplemented with 200 μL DPPH. Then, incubation was done at 37°C for 30 min. Finally, the absorbance was read at 517 nm and distilled water was used as a control (Das & Goyal, 2015).

To evaluate hydroxyl radical scavenging activity (HRSA), a mixture containing 1‐mL ferrous sulfate (0.75 mM), 2‐mL sodium phosphate buffer (pH 7.4), 1‐mL hydrogen peroxide (0.12%, v/v), and 1 mL of 1,10‐phenanthroline (0.75 mM) was added to a 1‐mL juice sample. Subsequently, the incubation was performed at 37°C for 90 min. Finally, the absorbance of the mixture was read at 536 nm (Zhang et al., 2021).

For the ferrous reducing power (FRP) assay, a 0.05 mL of juice sample was supplemented with 0.025 mL of phosphate buffer and 0.025 mL of potassium ferricyanide, and then incubation was carried out at 37°C for 60 min. Afterward, the mixture was supplemented with 0.025 mL of trichloroacetic acid (10%) and 0.1 mL of distilled water, and the absorbance was measured at 700 nm. Finally, 0.025 mL of ferric chloride (0.1%) was added to the mixture, and the absorbance was determined again (Kuda et al., 2016).

### 2.8 Enzyme assays

For the α‐amylase inhibition assay, 50‐μL sample was added to 50‐μL α‐amylase (0.2 U/mL), and incubation was done at 25°C for 10 min in 96‐well. Each well was supplemented with 50 μL of 1% soluble starch solution, and then incubation was carried out again at 25°C for 10 min. Afterward, 100‐μL dinitrosalicylic acid color reagent was added, and a boiling water bath was used to stop the reaction. The solutions were diluted to proper multiple with distilled water. Finally, the absorbance of each well was read at 540 nm and the distilled water was used as a control (Zhang et al., 2021).

To evaluate the α‐glucosidase inhibition test, 50 μL of the sample was added to 100‐μL α‐glucosidase (1 U/mL) subsequently, incubation was performed at 37°C for 10 min in 96‐well. Then, each well was supplemented with 50‐μL p‐nitrophenyl‐α‐glucopyranoside (5 mmol/L) and kept at 37°C for 5 min to perform the test. The absorbance was determined at 405 nm, and distilled water was used as a control (Dang et al., 2018).

For angiotensin‐converting enzyme **(**ACE) inhibition assay, 100 μL of ACE (25 mU/mL) solution and 40‐μL samples produced at various concentrations were kept at 37°C for 15 min. To initiate the enzymatic reaction, 100 μL of 8.33 mmol/L substrate, Hip‐His‐Leu, and 0.3 mol/L NaCl in 50 mmol/L sodium borate buffer (pH 8.3) was added to the solution. Then, incubation was carried out at 37°C for 15 min, and the reaction was ended by adding 150 μL of 1 M HCl. Each tube was supplemented with 1000 μL of ethyl acetate, and subsequently, centrifugation was done at 2000 *g* for 15 min. After the evaporation of ethyl acetate layer, the residue was redissolved in distilled water, and its absorbance was monitored at 228 nm (Oboh et al., 2012).

### 2.9 Anti‐inflammatory activity

For the anti‐inflammatory assay, a 96‐well plate was supplemented with 150‐μL phosphate buffer, 20‐μL sample, 60‐μL linoleic acid, and 20‐μL 5‐LOX enzyme for measurement of 5‐ LOX enzyme activity. Subsequently, the mixture was kept at 25°C for 15 min, and the absorbance was monitored at 234 nm (Villarreal‐Soto et al., 2019).

### 2.10 Sensory analysis

Sensory analysis was done using a 24‐member trained panel, in which the panelists scored the overall acceptability using a 9‐point hedonic scale. Number 1 had the lowest score, and number 9 had the highest (Hashemi et al., 2016).

### 2.11 Statistical analysis

All tests were carried out in triplicate. Analysis of variance (ANOVA) and Duncan were used (SPSS package program; v. 20.0), and values of *p* < .05 were considered as statistically significant.

## 3 RESULTS AND DISCUSSION

### 3.1 Viable counts of *Lactobacillus* strains

**FIGURE 1:**
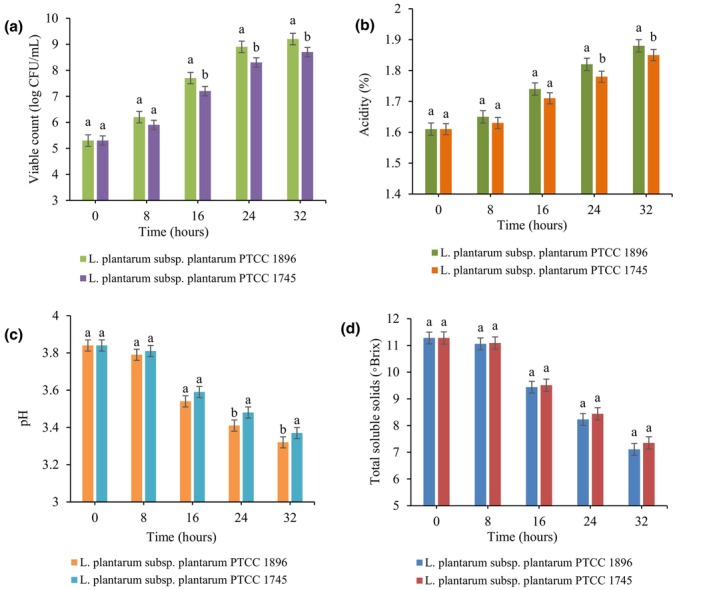
Changes in viable count (a), acidity (b), pH (c), and total soluble solids (d) in guava juice fermented with *L. plantarum* subsp. *plantarum* PTCC 1896 and *L. plantarum* subsp. *plantarum* PTCC 1745. Error bars reveal the standard deviation of each measurement in guava juice during 32 h of fermentation. Values with different lowercase letters are significantly different within the same fermentation hour (*p <* .05).

Viable counts of both *Lactobacillus* strains (*L. plantarum* subsp. *plantarum* PTCC 1896 and *L. plantarum* subsp. *plantarum* PTCC 1745) in guava juice were evaluated during 32 h of fermentation (Figure 1a). According to the figure, the population of both strains was 5.3 logs CFU/mL at zero time, which significantly (*p <* .05) increased with the elapse of fermentation time. Guava juice is a source of nondigestible carbohydrates and simple sugars, which can be a suitable substrate for *Lactobacillus* strains to grow well in this medium (de Oliveira et al., 2020). Similarly, Liao et al. (2016) indicated that the number of *L. plantarum* J26 in blueberry juice reached 9.12 log CFU/mL after 24 h of fermentation. Moreover, the results revealed that the growth of *L. plantarum* subsp. *plantarum* 1896 was notably (*p <* .05) higher than *L. plantarum* subsp. *plantarum*1745, and they reached viable counts of 9.2 and 8.8 log CFU/mL, respectively, at the end of the fermentation time. This difference in growth can be due to the difference in the physiological characteristics of bacteria and the growth medium (Hashemi & Jafarpour, 2020).

### 3.2 Physicochemical properties

The trend of acidity and pH changes during fermentation is presented in Figure 1b,c. As can be observed in the figure, the pH of guava beverage decreased significantly (*p <* .05) during the fermentation period, showing the acidity the opposite trend. Moreover, the Brix of the fruit juice decreased continuously throughout the fermentation period (Figure 1d). These results are related to the increase in bacterial growth during the fermentation process (Section 3.1), which leads to an enhancement in their metabolic activities, including consumption of reducing sugars and production of organic acids (Mousavi et al., 2011). According to the data, *L. plantarum* subsp. *plantarum* 1896 led to more changes in acidity and pH compared to *L. plantarum* subsp. *plantarum*1745, so the initial values reached from 1.61% and 3.84% to 1.88% and 3.32%, respectively, after 32‐h fermentation. Similar findings were reported for the acidity and pH of carrot juice fermented by *Bifidobacterium* strains (Kun et al., 2008). In addition, a decrease in TSS content and pH was observed due to the metabolism of sugars during the fermentation of noni juice (Wall et al., 2015).

### 3.3 Consumption of sugars

**FIGURE 2:**
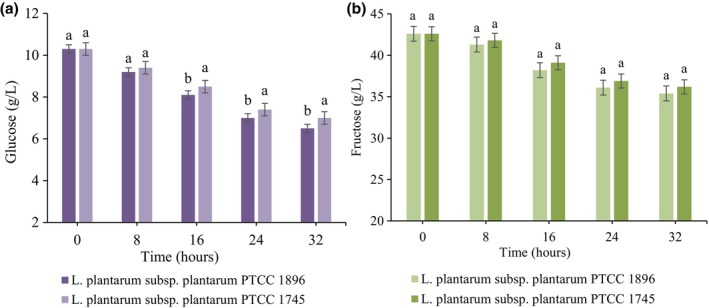
Variations in glucose (a) and fructose (b) content in guava juice fermented with *L. plantarum* subsp. *plantarum* PTCC 1896 and *L. plantarum* subsp. *plantarum* PTCC 1745. Error bars reveal the standard deviation of sugar content in guava juice during 32 h of fermentation. Values with different lowercase letters are significantly different within the same fermentation hour (*p <* .05).

The metabolism of glucose and fructose during 32 h of guava juice fermentation is illustrated in Figure 2. As can be seen, both studied *Lactobacillus* strains consumed the sugars and the amounts of both carbohydrates significantly (*p <* .05) decreased during the fermentation time. The improved growth of bacterial cells may be due to the better consumption of culture medium, higher availability of reducing sugars, and increased production of organic acids. This can cause a further decrease in pH and less available remaining simple sugars (Mousavi et al., 2013). Moreover, in several studies, glucose has been introduced as the main carbohydrate source for *Lactobacillus* strains (Jahandideh et al., 2012; Kun et al., 2008). According to the data, it was found that there is a significant difference (*p <* .05) between the two *Lactobacillus* strains in terms of glucose consumption. A lower level of glucose residues was detected for *L. plantarum* subsp. *plantarum* 1896 compared to *L. plantarum* subsp. *plantarum*1745. The initial amount of glucose (10.3 g/L) reached 6.5 g/L and 7 g/L for *L. plantarum* subsp. *plantarum* 1896 and *L. plantarum* subsp. *plantarum*1745, respectively, after 32‐h fermentation. On the other hand, no significant differences (*p >* .05) were observed between *L. plantarum* subsp. *plantarum* 1896 and *L. plantarum* subsp. *plantarum*1745 in terms of fructose consumption. It has been demonstrated that the amount of sugar consumption by *Lactobacillus* depends on several factors, including the bacterial strain, the type of carbohydrate, and the fermentation time (Hou et al., 2000). In a previous study, we observed that the amount of glucose and fructose markedly decreased during the fermentation of bergamot juice with *Lactobacillus* strains, but the amount of carbohydrate consumption was different depending on the type of treatment (pure or mixed cultures) and the type of sugar (Hashemi & Jafarpour, 2020).

### 3.4 Changes in antioxidant bioactive compounds

**FIGURE 3:**
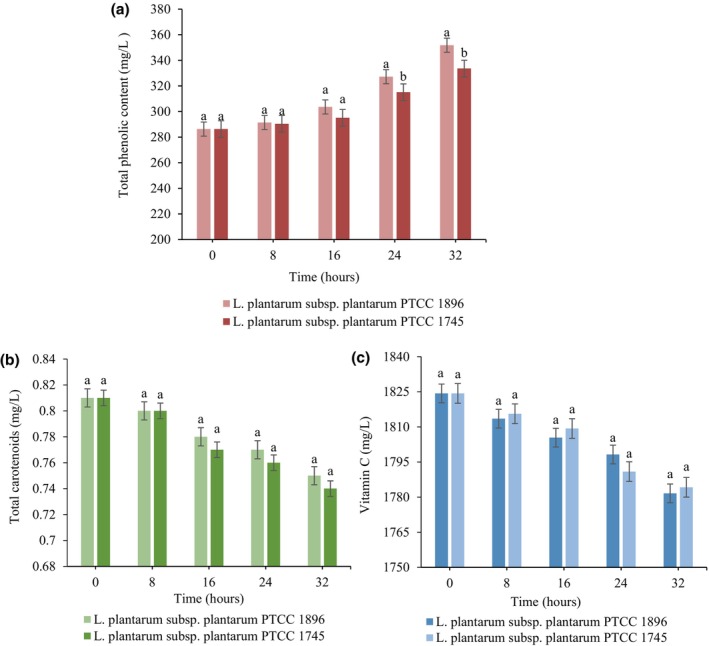
Changes in total phenolic content (a), total carotenoids (b), and vitamin C (c) content in guava juice fermented with *L. plantarum* subsp. *plantarum* PTCC 1896 and *L. plantarum* subsp. *plantarum* PTCC 1745. Error bars reveal the standard deviation of each measurement in guava juice during 32 h of fermentation. Values with different lowercase letters are significantly different within the same fermentation hour (*p <* .05).

As shown in Figure 3a, TPC increased notably in treated juices during fermentation (*p <* .05). In addition, fermentation with *L. plantarum* subsp. *plantarum* 1896 had a more pronounced effect on the level of TPC than *L. plantarum* subsp. *plantarum*1745, in which the initial amount of TPC (286.3 mg/L) was increased between 18% and 23%, and after 32 h of fermentation with *L. plantarum* subsp. *plantarum* 1745 and *L. plantarum* subsp. *plantarum*1896, respectively. Overall, fermentation led to the hydrolysis of glycosyl phenolic compounds and the production of free phenolic compounds, playing the production of enzymes such as *β*‐glucosidase by fermentative bacteria an important role in creating such complex changes (de Oliveira et al., 2020). For instance, previous researches have demonstrated an increase in TPC in fruit juices fermented with *Lactobacillus* strains (Kwaw et al., 2018; Zhang et al., 2021).

On the other hand, the data obtained for total carotenoid content (TCC) changes in guava juice containing *L. plantarum* subsp. *plantarum* 1896 and *L. plantarum* subsp. *plantarum*1745 indicated a decreasing trend in carotenoid levels at different times of fermentation. According to Figure 3b, fermentation with *L. plantarum* subsp. *plantarum*1896 and *L. plantarum* subsp. *plantarum*1745 resulted in 8% and 9.46% reduction in TCC of juice samples at the end of the fermentation time, respectively. The available literature shows contrasting results regarding the behavior of carotenoids after fermentation (Mapelli‐Brahm et al., 2020). Similar to our study, Kun et al. (2008) found a 15–45% degradation in carrot juice carotenoids inoculated with Bifidobacterium strains. However, de Oliveira et al. (2020) showed that 120‐h fermentation of guava by‐products with selected *Lactobacillus* isolates *did not alter the TCC*. Degradation of carotenoids may be due to microbial metabolism, while other factors such as fermented matrix, pH, and temperature of fermentation can be considered as effective factors (Panda & Ray, 2007).

Variations in the vitamin C content of guava juice during fermentation are depicted in Figure 3c. Data showed a significant (*p <* .05) decreasing trend in vitamin C with time of fermentation; however, no significant (*p >* .05) differences between the two juices treated with *L. plantarum* subsp. *plantarum*1896 and *L. plantarum* subsp. *plantarum*1745 were found. Retention of ~98% of the initial amount of vitamin C (1824.3 mg/L) was observed after 32‐h fermentation with both *L. plantarum* subsp. *plantarum*1896 and *L. plantarum* subsp. *plantarum*1745. These findings confirm with the results of Panda et al. (2017), who indicated a decrease in vitamin C in prickly pear juice after fermentation by *Limosilactobacillus fermentum*. This reduction may be related to the increment in the activity of the ascorbate oxidase enzyme produced by fermenting microorganisms (Hashemi & Jafarpour, 2020). However, researchers demonstrated that fermentation can have a heterogeneous effect on the amount of vitamin C in fermented fruit juices (Szutowska, 2020). Kaprasob et al. (2017) showed that the content of vitamin C in cashew apple juice increased after 12 h of fermentation with *L. plantarum* and remained stable after 48 h, while fermentation with *Lactobacillus acidophilus* led to a decrease in vitamin C content after 48 h of fermentation. This shows that the amount of vitamin C and its stability in the fruit juice during fermentation depends on various factors, including pH of the medium, mineral concentration, the redox state, sugars, and the presence of metal ions (Hashemi et al., 2017).

### 3.5 Antioxidant activity

**FIGURE 4:**
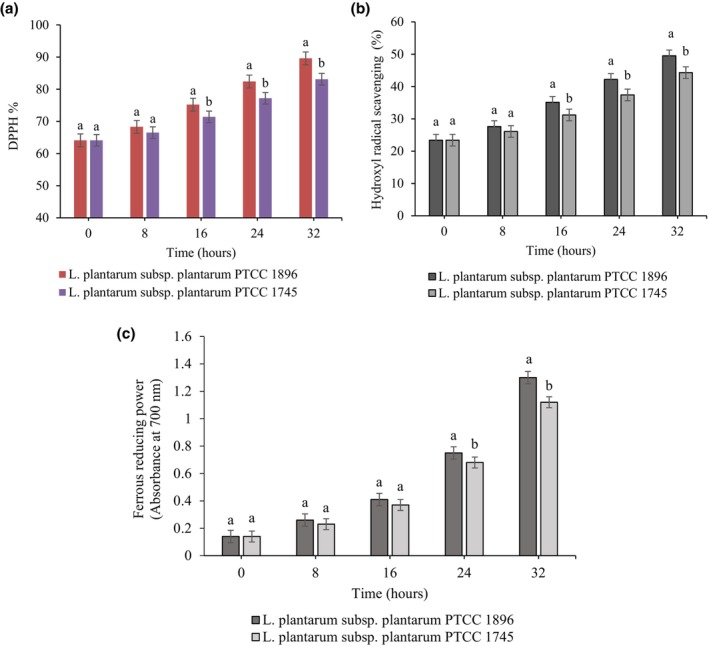
Changes in antioxidant activity of guava juice fermented with *L. plantarum* subsp. *plantarum* PTCC 1896 and *L. plantarum* subsp. *plantarum* PTCC 1745. Error bars reveal the standard deviation of each measurement in guava juice during 32 h of fermentation. Values with different lowercase letters are significantly different within the same fermentation hour (*p <* .05).

Three antioxidant indexes were used to evaluate the antioxidant activity of fermented guava juice samples, including DPPH, HRSA, and FRP. According to Figure 4, the fermentation process with the studied *Lactobacillus* strains exhibited a significant increase (*p <* .05) in the antioxidant activity of the juice samples and treatment with *L. plantarum* subsp. *plantarum*1896 showed more radical scavenging ability and reducing power activity than *L. plantarum* subsp. *plantarum*1745. After 32‐h fermentation with *L. plantarum* subsp. *plantarum*1896 and *L. plantarum* subsp. *plantarum*1745, the amount of DPPH increased from 64.1% to 89.6% and 83.1%, and the initial level of HRSA (23.4%) increased up to 49.5% and 44.3%, respectively (Figure 4a,b). Typically, DPPH assay is used to examine the hydrogen‐donating ability of antioxidants to scavenge the DPPH as a free radical. Hydroxyl (OH˙) radicals are the main reactive oxygen species that cause enormous damage to biological molecules (Rahman et al., 2015). Therefore, the HRSA assay was used to measure the potential of fermented juices to trap the OH˙radicals generated by the Fe^3+^–EDTA–ascorbate–H_2_O_2_ system. The current increasing trend of free radical scavenging ability is in line with the reports of Nguyen et al. (2019) and Mousavi et al. (2013), who indicated an increment in the antioxidant activity of fruit juices during fermentation with *Lactobacillus* strains.

The reducing power of fermented guava juice was also examined. Generally, the reducing power is related to the presence of reductants, which break the free radical chain by donating hydrogen atoms (Rahman et al., 2015). In the present study, the reduction amount of Fe^3+^ (ferric iron) to Fe^2+^ (ferrous iron) in the juice samples was investigated. As shown in Figure 4c, the absorbance of juices fermented with *L. plantarum* subsp. *plantarum* 1896 and *L. plantarum* subsp. *plantarum*1745 was 0.14 at zero time and increased to 1.3 and 1.12 after 32 h of fermentation, respectively. Similarly, an increase in FRAP of peach juice fermented with *L. acidophilus* and *L. fermentum* during the fermentation period has been reported (Hashemi et al., 2021). In light of the above considerations, data showed that the fermented guava juice exhibited a proper OH˙and DPPH radical scavenging activity along with the ferrous reducing ability, which can be used as a good source of antioxidants. Several researches have indicated a linear correlation between TPC and antioxidant activity in vegetables and fruits (de Oliveira et al., 2020; Zhang et al., 2021). The antioxidant activity of phenolic compounds is basically due to their chemical structure and redox properties, in which hydroxyl groups act as free radical neutralizing and adsorbing agents (Kuda et al., 2016). Furthermore, LAB metabolites produced during fermentation may act as reductones and react with free radical species (Cheng et al., 2020). Our findings are consistent with the report of Zhao and Shah (2014), who demonstrated that the enhancement in antioxidant activity and reducing power of soymilk is related to the intrinsic antioxidants and also electron‐donating compounds such as bioactive peptides and isoflavone aglycones produced by selected *Lactobacillus* species during fermentation.

### 3.6 Enzyme inhibition ability, ACE inhibitory, and anti‐inflammatory activities

**FIGURE 5:**
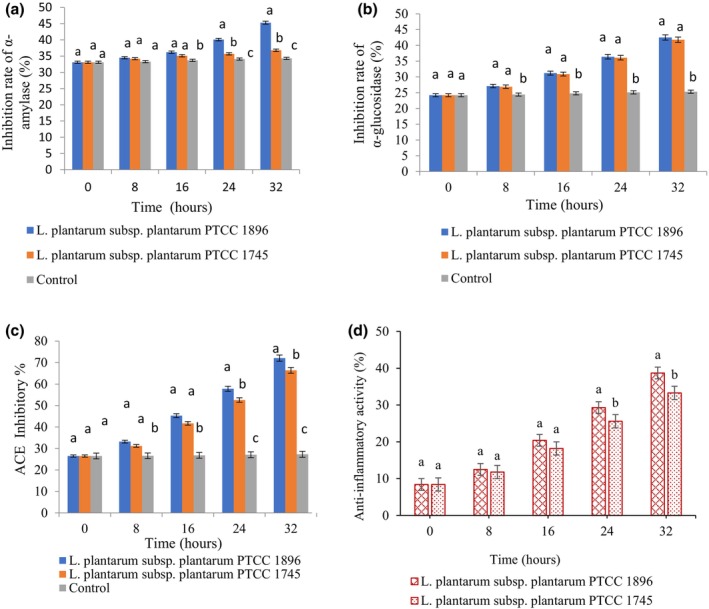
Anti‐hyperglycemic (a, b), anti‐hypertensive (c), and anti‐inflammatory (d) activities of guava juice fermented with *L. plantarum* subsp. *plantarum* PTCC 1896 and *L. plantarum* subsp. *plantarum* PTCC 1745. Error bars reveal the standard deviation of each measurement in guavajuice during 32 h of fermentation. Values with different lowercase letters are significantly different within the same fermentation hour (*P* <0.05).

In addition to the basic nutritional features of fermented fruit and vegetable juices, they have been found to have other health effects, such as anti‐inflammatory, antihypertensive, and antidiabetic properties (Szutowska, 2020). In the present study, α‐amylase and α‐glucosidase were evaluated to investigate the antihyperglycemic impact of fermented guava juice. α‐amylase and α‐glucosidase are two significant enzymes in the human digestive tract that hydrolyze polysaccharides in food and turn them into simple sugars that the human body can absorb. The inhibition of these two enzymes plays a vital role in reducing the absorption of dietary sugar in the human digestive tract (Oboh et al., 2012). According to Figure 5a,b, the inhibition of both mentioned enzymes significantly (*p <* .05) increased at the end of the fermentation time. In addition, fermentation with *L. plantarum* subsp. *plantarum* 1896 showed a higher (*p <* .05) inhibition rate of α‐amylase than *L. plantarum* subsp. *plantarum*1745, but no significant differences (*p >* .05) were observed between the α‐glucosidase inhibitory activities of the studied bacteria. The inhibition of digestion and absorption of dietary carbohydrates is one of the main therapeutic approaches to control postprandial hyperglycemia. Recently, studies have demonstrated that some phytochemicals, such as polyphenols, terpenes, flavonoids, and saponins, can inhibit the activity of digestive enzymes (Nayak et al., 2010). For instance, Zhang et al. (2021) indicated that fermented blueberry juice had a higher enzyme inhibition rate than nonfermented juice. They stated that the increase in the production of phenolic compounds and the bio‐transformation of active substances by the *Lactobacillus* strain are the main reasons for this increase in the fermented product. Moreover, the antihyperglycemic effect of fermented bitter melon juice has been reported, which was associated with a greater content of functional substances such as aglycones and other phenolic phytochemicals (Mazlan et al., 2015).

The ACE inhibitory activity of guava juice during fermentation is depicted in Figure 5c. The inhibitory activity of guava juice notably increased throughout the entire fermentation time (*p <* .05). Fermentation with *L. plantarum* subsp. *plantarum*1896 and *L. plantarum* subsp. *plantarum*1745 promoted a 2.72‐ and 2.5‐fold increase in the antihypertensive functionality of the produced beverage after 32 h of fermentation, respectively. Hypertension or high blood pressure is a cardiovascular disease that can cause damage to the heart, brain, and blood vessels. ACE is a metalloenzyme that plays an important role in regulating blood pressure in the human body. This enzyme is responsible for converting angiotensin I to active angiotensin II. Indeed, angiotensin II stimulates the secretion of aldosterone from the adrenal glands, which increases blood pressure by reabsorption of sodium. Since ACE plays a critical role in homeostasis, blood pressure, and retention of water and electrolytes, it is considered an important factor in treating blood pressure and heart failure (Oboh et al., 2012). Consistent with our results, Simsek et al. (2014) observed an increase in ACE inhibitory activity in fermented vegetable juice and attributed this increase to the synthesis of bioactive peptides during fermentation. Studies have shown no direct relationship between the amount of bioactive peptides and inhibitory functionality, so these peptides can exert their inhibitory effect even in small amounts (Chen et al., 2015). Moreover, some reports associate ACE inhibitory activity with the inhibitory capacity of flavonoids (Oboh et al., 2012). Guava fruit is a rich source of flavonoids, anthocyanins, and triterpenoids, and the ACE inhibitory activity of the fermented beverage may be related to these phytochemical compounds (Flores et al., 2015).

The human body's inflammatory response occurs by releasing certain chemicals, such as leukotrienes. The 5‐lipoxygenase (5‐LOX) enzyme synthesizes this substance from arachidonic acid. This enzyme's inhibitors are considered analgesic and anti‐inflammatory compounds (Villarreal‐Soto et al., 2019). In the present study, the anti‐inflammatory potential of fermented guava juice was investigated through the inhibitory action on 5‐LOX. According to Figure 5d, the anti‐inflammatory activity of juice samples was markedly enhanced during the fermentation process (*p <* .05). Fermentation with *L. plantarum* subsp. *plantarum*1896 showed a higher inhibition rate of 5‐LOX than *L. plantarum* subsp. *plantarum*1745, and the anti‐inflammatory activity of fermented samples was achieved 4.6 and 3.9 times higher than unfermented fruit juice, respectively, after 32 h of fermentation. Our results are consistent with the previous work, in which the fermentation of peach juice with selected *Lactobacillus* strains resulted in increased anti‐LOX activity (Hashemi et al., 2021). Researches have shown that metabolic substances such as free phenolics, natural acids, and bacteriocins are produced during fermentation with LAB, which have potent anti‐inflammatory properties (Carvalho et al., 2017; Hur et al., 2014). In addition, it has been indicated that increased anti‐inflammatory properties are associated with increased antioxidant activities in fermented pomegranate juice (Filannino et al., 2013).

### 3.7 Sensory evaluation

**FIGURE 6:**
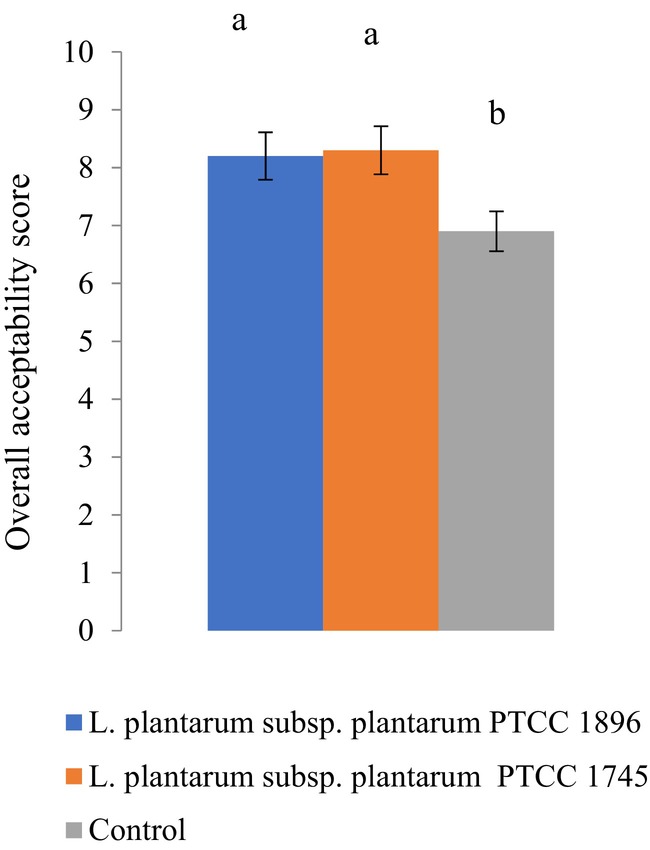
Overall acceptability of guava juice fermented with *L. plantarum* subsp. *plantarum* PTCC 1896 and *L. plantarum* subsp. *plantarum* PTCC 1745. Values with different lowercase letters are significantly different (*p <* .05).

The sensory evaluation results (Figure 6) showed that the fermentation process increased the overall acceptability of fruit juice compared to the nonfermented samples (controls). No significant difference was observed between the fermented samples with *Lactobacillus* strains. The fermentation process can improve fruit juice flavor and increase consumers' acceptance (Rajendran et al., 2023).

## 4 CONCLUSIONS

According to the achieved data, it can be concluded that fermentation of guava juice with the studied *Lactobacillus* strains notably affected the physicochemical features of the produced beverage. Moreover, the fermentation process markedly improved the antioxidant activity and TPC in the final product. The present work indicated that the fermented fruit juice had health‐beneficial properties, including low simple sugar content, α‐glucosidase, and α‐amylase inhibitory activities, antihypertensive, and anti‐inflammatory properties. Results revealed that *L. plantarum* subsp. *plantarum* 1896 was more effective than *L. plantarum* subsp. *plantarum*1745 on the functional characteristics of fermented juice samples. Therefore, according to the nutritional value and bioactive potential of fermented guava juice, it can be utilized as a nondairy functional beverage by vegetarians and people who are lactose or milk protein intolerant. However, further studies can be done to investigate the optimal fermentation conditions to ameliorate the bioactive properties of the final product. Besides, in‐vivo experiments are recommended to evaluate the clinical impact of the produced fermented beverage.

## AUTHOR CONTRIBUTIONS

**Seyed Mohammad Bagher Hashemi:** Conceptualization (equal); supervision (equal); writing – review and editing (equal). **Dornoush Jafarpour:** Formal analysis (equal); investigation (equal); writing – original draft (equal); writing – review and editing (equal).

## FUNDING INFORMATION

This research did not receive any specific grant.

## CONFLICT OF INTEREST STATEMENT

The authors declare that they have no conflict of interest.

## ETHICS STATEMENT

This study does not include any animal or human testing.

## CONSENT TO PARTICIPATE

All the co‐authors participated in the preparation of this manuscript.

## CONSENT FOR PUBLICATION

All authors have read and agreed to the published version of the manuscript. All authors read and approved the final manuscript.

## Data Availability

Data are not shared.
